# Intramedullary nail holes laser indicator, a non-invasive technique for interlocking of intramedullary nails

**DOI:** 10.1038/s41598-021-00382-8

**Published:** 2021-10-27

**Authors:** Mohammadreza Maleki, Alireza Fadaei Tehrani, Ayda Aray, Mehdi Ranjbar

**Affiliations:** 1 Department of Mechanical Engineering, Isfahan University of Technology, 84156-83111 Isfahan, Iran; 2 Department of Physics, Isfahan University of Technology, 84156-83111 Isfahan, Iran

**Keywords:** Optical physics, Optics and photonics, Applied optics, Lasers, LEDs and light sources, Optical physics, Optical techniques, Anatomy, Bone, Medical research, Outcomes research, Bone quality and biomechanics

## Abstract

Interlocking of intramedullary nails is a challenging procedure in orthopedic trauma surgery. Numerous methods have been described to facilitate this process. But they are exposed patient and surgical team to X-rays or involves trial and error. An accurate and non-invasive method has been provided to easily interlocking intramedullary nails. By transferring a safe visible light inside the nail, a drilling position appears which use to drilling bone toward the nail hole. The wavelength of this light was obtained from ex-vivo spectroscopy on biological tissues which has optimal transmission, reflectance, and absorption properties. Moreover, animal and human experiments were performed to evaluate performance of the proposed system. Ex-vivo performance experiments were performed successfully on two groups of cow and sheep samples. Output parameters were procedure time and drilling quality which there were significant differences between the two groups in procedure time (P < 0.05). But no significant differences were observed in drilling quality (P > 0.05). Moreover, an In-vivo performance experiment was performed successfully on a middle-aged man. To compare the provided method, targeting-arm, and free-hand techniques, two human experiments were performed on a middle-aged and a young man. The results indicate the advantage of the proposed technique in the procedure time (P < 0.05), while the drilling quality is equal to the free-hand technique (P = 0.05). Intramedullary nail holes laser indicator is a safe and accurate method that reduced surgical time and simplifies the process. This new technology makes it easier to interlocking the intramedullary nail which can have good clinical applications.

## Introduction

Intramedullary nailing is one of the most common surgical operations for fixation of long bone fractures. Nail deformation affects the accuracy of distal interlocking of the intramedullary nails (IMN) which is a common procedure in tibia and femoral fractures^1–4^. Moreover, holes locations of the intramedullary nail are unknown which adds to the deformation problem^5–9^. Numerous techniques for interlocking screw insertion have been proposed to optimize number of drilling and operation time and avoid ionizing radiation exposure^10,11^. However, these techniques have not gained widespread clinical application. Other methods are based on fluoroscopy that called free-hand technique (FH) or based on a special device designed for this purpose, knows as targeting-arm devices (TAD)^12–15^. Fluoroscopy is a high-cost operation in which patients and the surgical team are exposed to X-ray. Targeting device is a time-consuming process that involves trial and error^16,17^.

To reduce the high risk of X‐ray exposure, increases surgical accuracy, and accelerate the interlocking procedure, significant progress has been developed. The flag and grid technique increases the accuracy of intramedullary nailing by displaying the position of nail holes relative to the flag and grid in fluoroscopic images^18^. In addition to the risk of X-ray exposure, there is still the possibility of error due to the slipping and flag and grid displacement. Computer‐aided IMN localization techniques were also introduced which representing a significant reduction in fluoroscopic process time for interlocking screw insertion in a high-cost procedure^19–22^. Robotic techniques were also proposed in which a robotic system is utilized to drilling and installing fixation screws, requiring a large robot in the operating room representing a high cost, cumbersome, and X-ray-based method^23–25^. Naked‐eye 3D AR enables observation of the surgical path by costly and cumbersome equipment^26–29^. Electro-Magnetic (EM) and laser navigation systems can increase surgical accuracy without any X-ray exposure, but with excessive electrical noise causing large magnetic‐field distortion resulting in unstable tracking paths^30–38^. Moreover, the tracking accuracy is degraded with increasing distance of sensor and EM transmitter. This method is time-consuming and requires installation of various equipment^39^. Augmented fluoroscopy can decrease radiation exposure, surgical time, and simplify the distal interlocking process. But the patient and the surgical team are exposed to X-ray and it is a high-cost procedure^40–42^.

In this work, have been developed a non-invasive optical method for easy determining IMN holes position inside the bone canal utilizing a safe laser system with a safe visible wavelength that possesses proper transmittance, reflectance, and absorbance properties in human tissues. In order to find the optimized wavelength, Ex-Vivo spectroscopy experiments were performed on animal tissues i.e. on two groups of cow and sheep in the visible range of electromagnetic spectrum. Two parameters which are procedure time and drilling quality, are selected as the merit criteria which proves the acceptable performance of our proposed method. Then an In-Vivo performance experiment performed on a middle-aged man with a desired outcomes. To compare the provided method, targeting-arm, and free-hand techniques, two human experiments were performed on a middle-aged and a young man. The results indicate the advantage of the proposed technique. Intramedullary nail holes laser indicator is introduced here as a simple, non-invasive, and safe tool that reduces surgical time and simplifies the process. The presented Ex-Vivo and In-Vivo results pave the way for development of an effective and safe intramedullary nailing process in clinical applications.

## Materials and methods

**Figure 1:**
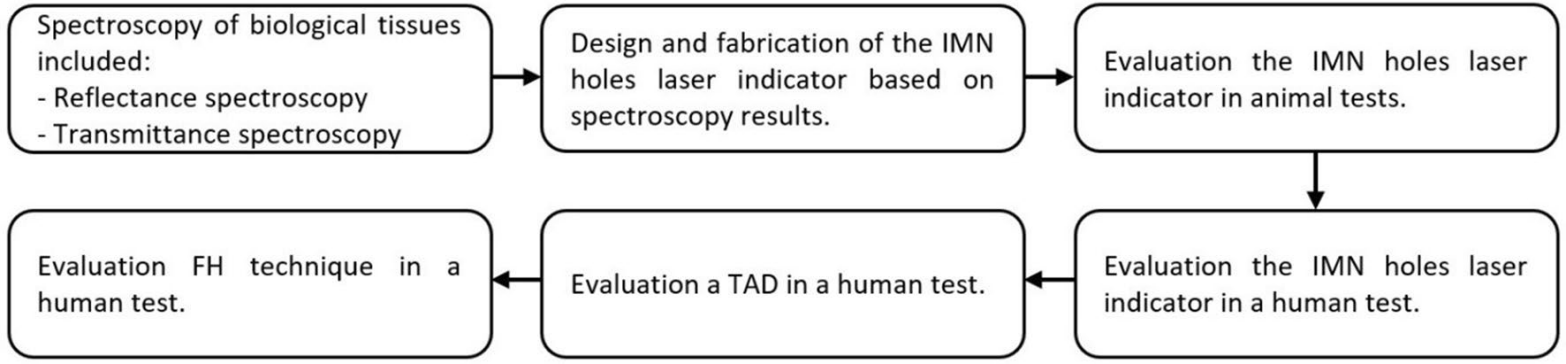
Flow diagram depicting the study selection procedure.

In this study have been proposed a novel radiation-free technique, using laser light to target the cross lock of the IMN. To achieve optimal wavelength, transmittance and reflectance spectroscopy of biological tissues were performed. Then, IMN holes laser indicator was made based on spectroscopy results. Animal and human tests were performed to evaluate the performance of the proposed technique. Figure 1 shows the study flow diagram.

### Spectroscopy of biological tissues

**Figure 2:**
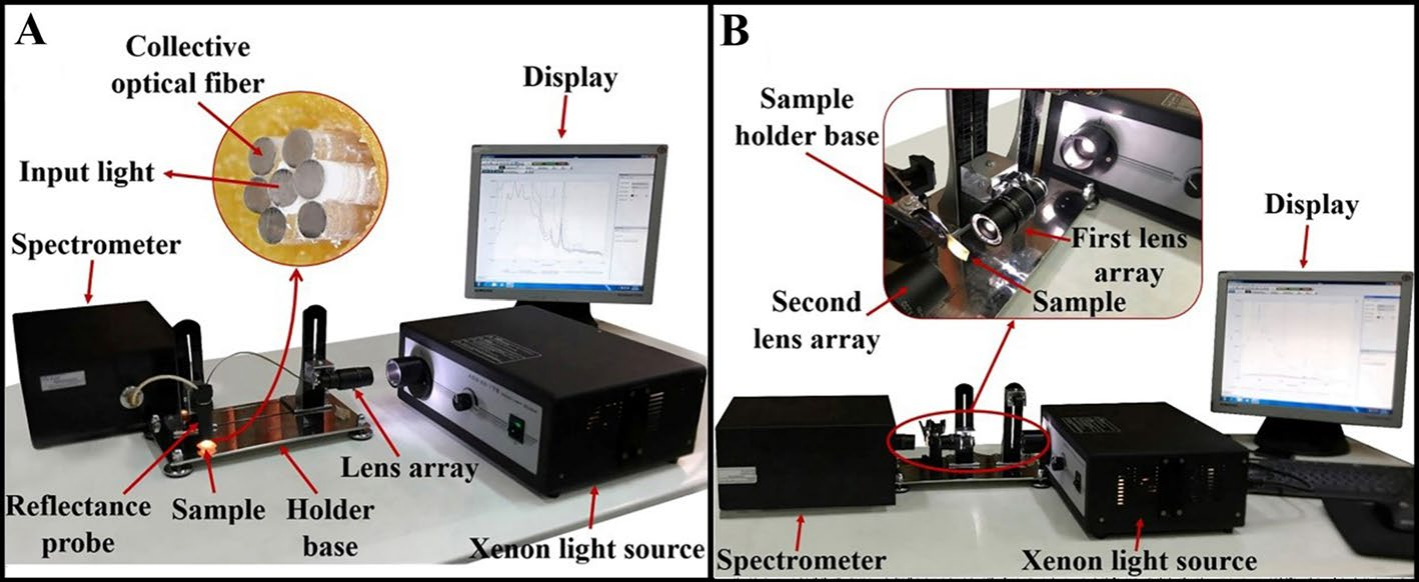
(**A**) Reflectance and (**B**) transmittance spectroscopy setups.

We performed Ex-Vivo spectroscopy of biological tissues including bone, fat, tendon, muscle, and skin. Samples were prepared in dimensions of 30 × 30 ± 2 mm and 2–4 mm thickness from cow and sheep legs which were supplied from the Zarrinshahr slaughterhouse, Iran. Two homemade spectroscopy setups were utilized: one for obtaining the reflectance spectrum (Fig. 2A) and another was designed for transmittance measurements (Fig. 2B). The reflectance measurements performed by a reflective probe mounted on a holder base which is placed at 5 mm hight from the sample. This reflective probe consists of seven optical fibres (750 µm, NA = 0.47 PMMA Optical Fibre-Mitsubishi Electric Co-Tokyo-Japan) which the middle one is coupled to the xenon source (ASB-XE-175-Spectral Products Co- Putnam-US) by a lens array. The induced light to the sample surface is collected by six side fibres and then the reflected spectrum is monitored by an optical spectrometer (Spectronix Ar 2015v- Teifsanje Co-Tehran-Iran). To eliminate the source spectral characteristics, a BaSO4 pill was used as a reference. We molded 4.49gr of BaSO4 powder (CAS Number: 7727-43-7-Titrachem Co-Iran) in 250mpa pressure. The reflectance spectrum of the homemade BaSO4 pill was collected and divided by the source spectrum yielding a flat spectral response through the visible spectrum.

Two lens arrays are utilized to obtain the transmittance spectrum, one mounted on the holder base for focusing the xenon light on the sample, and the second mounted in front of the first array to collect transmitted rays. The sample mounted on a sample holder in the proper distance from the lens arrays.

### IMN holes laser indicator

**Figure 3:**
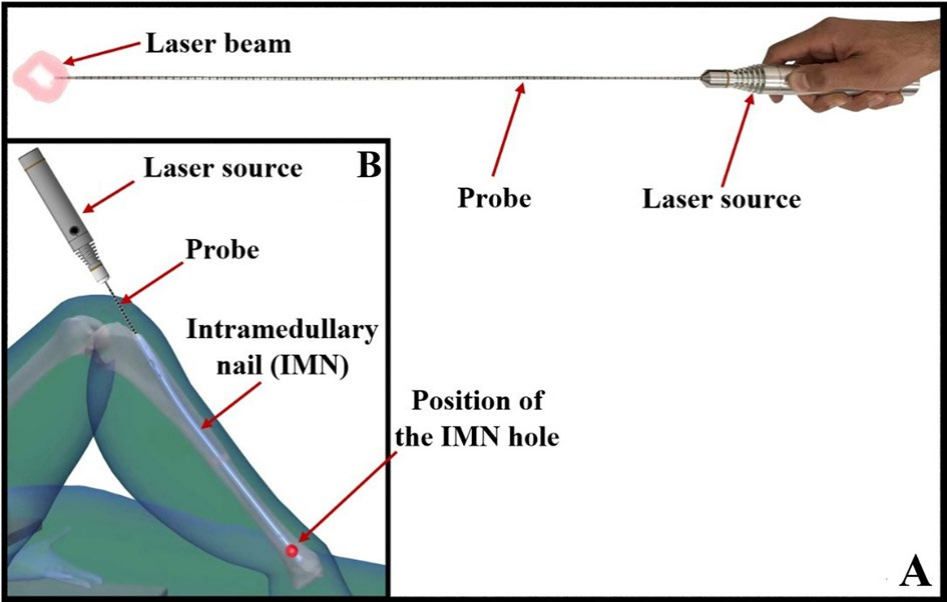
(**A**) IMN holes laser indicator and (**B**) a schematic image of positioning IMN hole using the IMN holes laser indicator.

As shown in Fig. 3A, the IMN holes laser indicator consists of a 680 nm–350 mW solid-state laser and a flexible biocompatible probe. The laser light is guided through the designed probe and then passes through the IMN hole, making the position of the IMN hole appears on the skin for the naked eyes. This portable device capable of washing, disinfecting, and autoclaving. Moreover, it uses a rechargeable lithium battery and can provide constant optical power during operation. Figure 3B shows a schematic image of positioning IMN hole using the IMN holes laser indicator.

### IMN holes laser indicator in animal tests

**Figure 4:**
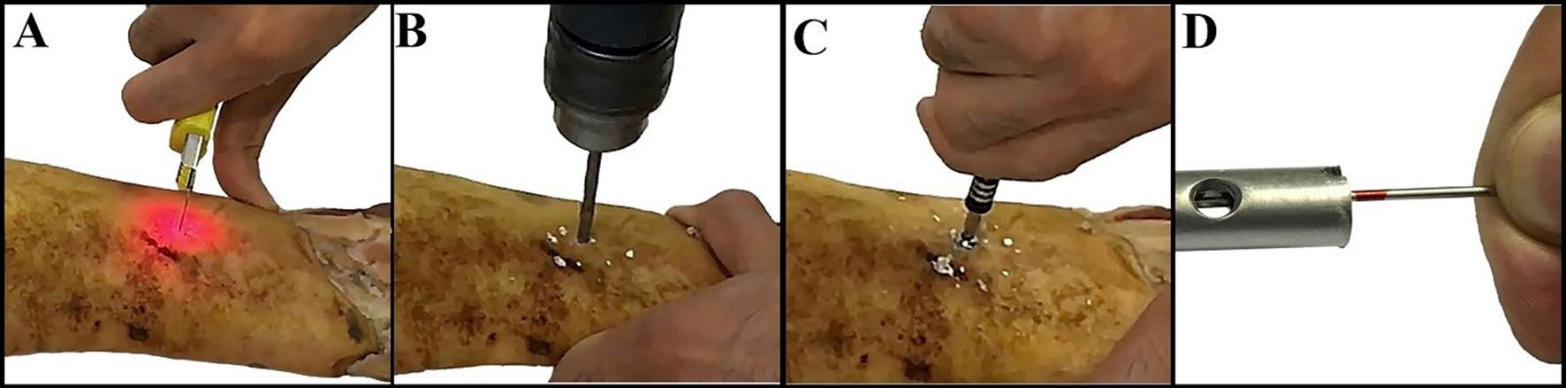
Intramedullary nailing for one of the samples, (**A**) determining the position of the IMN hole and skin incision, (**B**) bone drilling, (**C**) fastening the interlocking screw, (**D**) checking the screw insertion inside the IMN hole.

Measurements were performed on 10 animal samples, 5 sheep's legs, and 5 cows' legs which were supplied from the Zarrinshahr slaughterhouse, Iran. Sheep's samples had a small diameter between 16 and 21 mm and a large diameter between 29 and 35 mm. Also, the cows' samples had a small diameter between 40 and 48 mm and a large diameter between 65 and 73 mm. For each sample, intramedullary nailing (Pooyandegan Pezeshki Pardis-Golestan-Iran) was performed inside the tibia bone, and distal locking was done three times using the laser intramedullary nail holes indicator. Figure 4, shows intramedullary nailing procedures for one of the samples.

Output parameters were procedure time and drilling quality. The procedure time as one of the performance parameters is defined as the total time needed between determining the position of the IMN hole and checking the screw insertion. Another performance parameter is the drilling quality which is evaluated as follows: 3 points for successful operation and if the drill does not hit the nail, 2 points for successful operation but with a slight collision with the nail,1 point for severe interference of the drill with nail (in this case, the drilling site must be corrected), 0 point in case of failure. Mean value, standard deviation, and p-value were calculated for both the procedure time, and drilling quality. Paired t-tests were used to compare these performance parameters for cows' and sheep`s samples considering α = 0.05.

### IMN holes laser indicator in a human test

**Figure 5:**
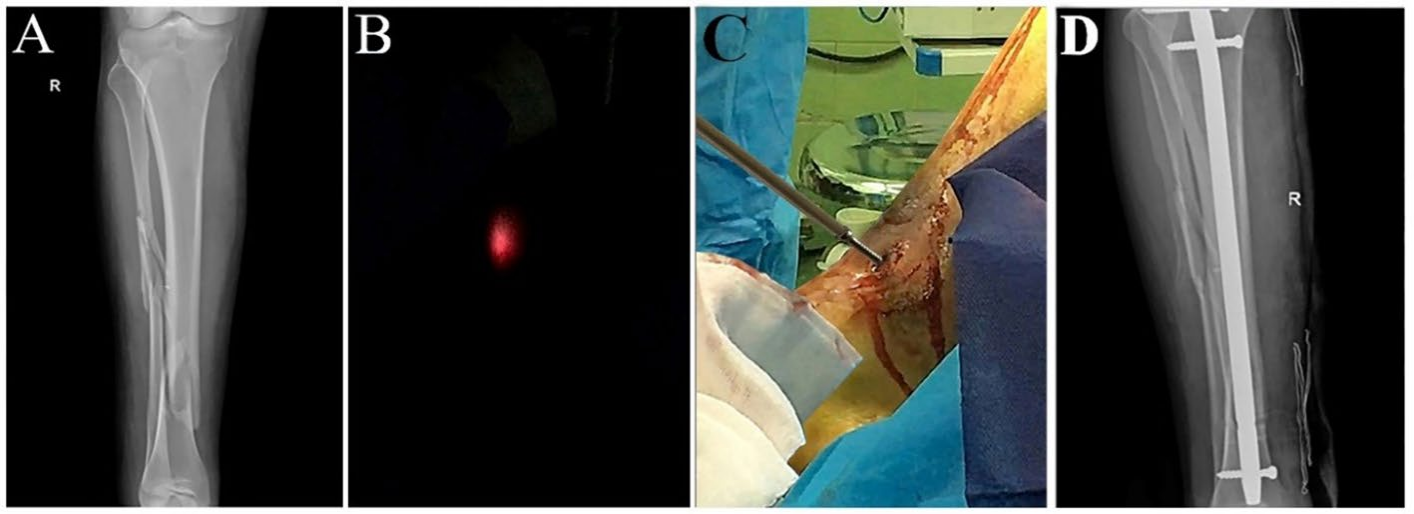
Interlocking of the intramedullary nail by the laser system, (**A**) X-ray image of the patient's right foot before the surgery, (**B**) Finding drilling position by the IMN holes laser indicator in the dark surgery room, (**C**) Interlocking the IMN, (**D**) The X-ray image of the patient's right foot one day after intramedullary nailing.

The evaluation was performed on a middle-aged man with an acute tibia and fibula fracture of the distal area of the right leg. The study was approved by the ethics committee of the Shohada Lenjan Hospital, Iran, in January 2020. Also, informed consent was obtained from participants and the study was performed under relevant guidelines and regulations. Figure 5A illustrates an X-ray image of the patient's right leg before the surgery.

Intramedullary nailing was performed using a tibia nail and three locking screws (Osveh Asia Medical Instrument Co—Iran). By utilizing the IMN holes laser indicator, finding drilling position, incisions skin, and drilling toward the IMN hole performed in a dark surgery room. Then, fastening the locking screw was performed in the natural light of the surgery room. Distal and proximal interlocking performed successfully for one hole in the distal area and two holes in the proximal area with no errors. Figure 5B finding drilling position by the IMN holes laser indicator in the dark surgery room, and Fig. 5C shows interlocking the IMN. Figure 5D shows the X-ray image of the right patient's foot one day after surgery.

### TAD in a human test

Interlocking was performed for the tibia bone of a middle-aged man with the left leg fracture using a TAD (Osveh Asia Medical Instrument Co—Iran). The study was approved by the ethics committee of the Shohada Lenjan Hospital, Iran, in September 2021. Also, informed consent was obtained from participants and the study was performed under relevant guidelines and regulations.

**Figure 6:**
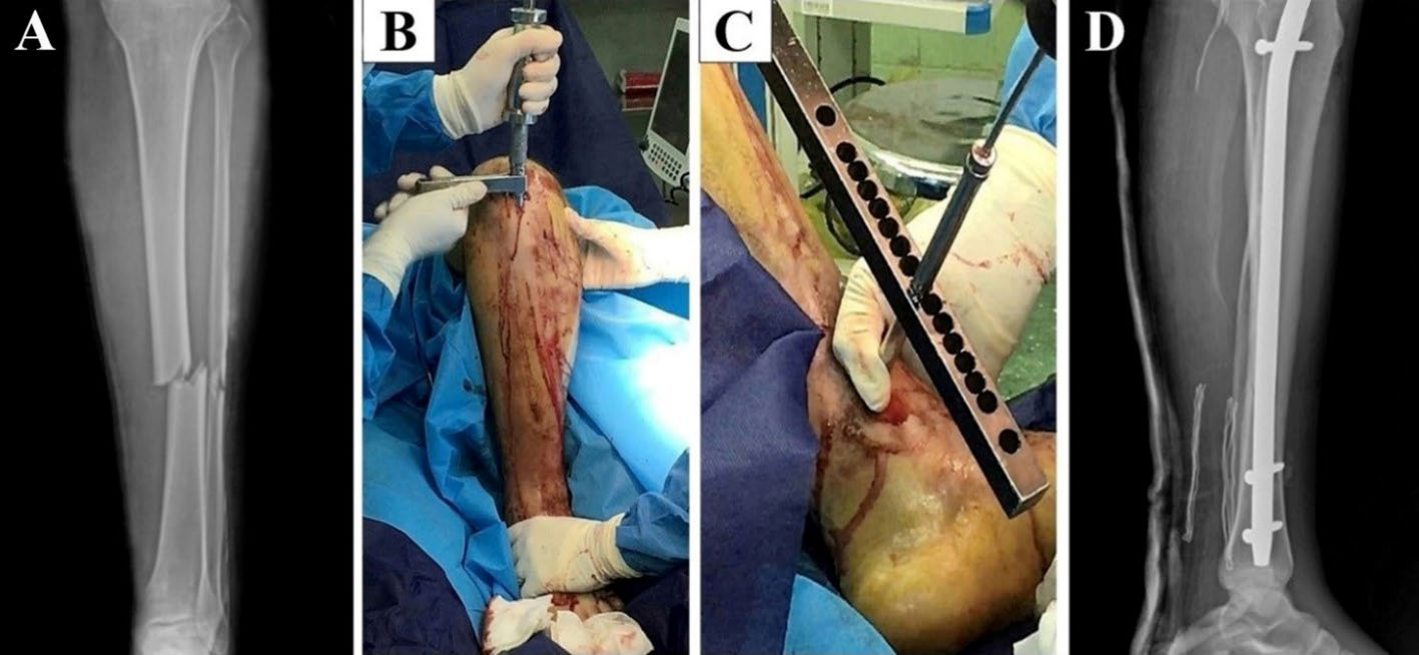
Interlocking of the intramedullary nail by the TAD, (**A**) an X-ray image of the patient's left foot before the surgery, (**B**) Inserting the IMN inside the tibia bone using the hammer, (**C**) Distal interlocking of the IMN by the TAD, (**D**) The X-ray image of the patient's left foot one day after surgery.

The IMN was fixed by three fixation screws. The distal interlocking process was performed by two locking screws which encountered errors while the proximal interlocking performed successfully. Figure 6A, illustrates an X-ray image of the patient's left leg before the surgery, Fig. 6B, inserting the IMN inside the tibia bone using a hammer, Fig. 6C, distal interlocking of the IMN by the TAD, and Fig. 6D, shows the X-ray image of the patient's foot one day after the surgery.

### FH technique in a human test

Interlocking of the IMN was done by a c-arm device (siemens healthineer-USA)**.** The patient was a young man with an acute tibia and fibula fracture of the distal area of the left leg. The surgery team was used X-ray protective lead clothes with a thyroid shield collar. The study was approved by the ethics committee of the Shohada Lenjan Hospital, Iran, in October 2021. Also, informed consent was obtained from participants and the study was performed under relevant guidelines and regulations.

**Figure 7:**
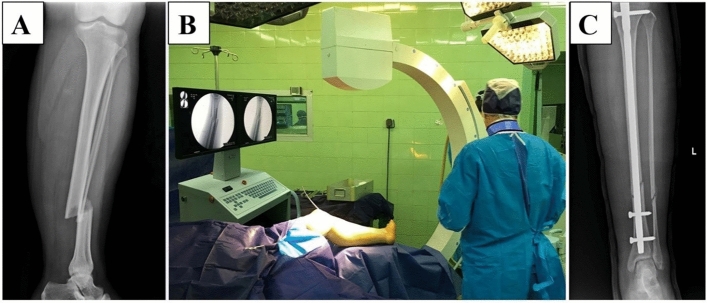
Interlocking of the intramedullary nail using FH technique, (**A**) The X-ray image of the patient's left foot before the surgery, (**B**) Inserted guide-wire inside the tibia bone under fluoroscopy control, (**C**) The X-ray image of the patient's left foot one day after the surgery.

Three fixation screws were used for the interlocking of the IMN. By controlling the movement direction of the drill under X-ray emission, no severe interference occurred between the drill and IMN. Figure 7A, illustrates an X-ray image of the patient's left foot before the surgery, Fig. 7B, inserted guide-wire inside the tibia bone under fluoroscopy control, and Fig. 7C, shows the X-ray image of the patient's left foot one day after the surgery.

## Results

### Spectroscopy of biological tissues

**Figure 8:**
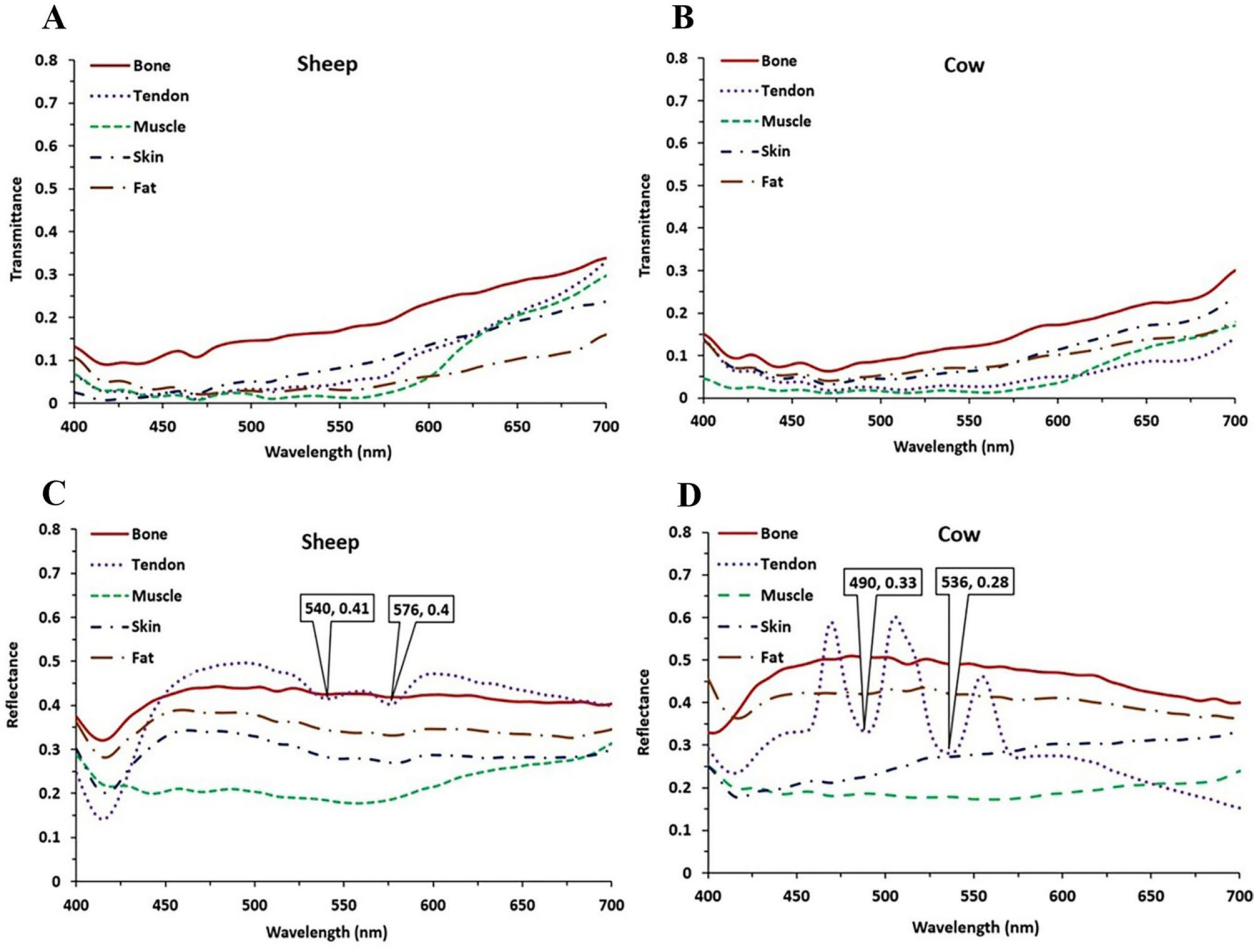
Spectral behavior of biological tissue in the range of 400 to 700 nm, transmittance spectrum of (**A**) sheep and (**B**) cow samples, reflectance spectrum of (**C**) sheep and (**D**) cow samples.

Ex-Vivo spectroscopy was performed on biological tissues in the visible range of electromagnetic spectrum. The optical properties of animal tissues of bone, tendon, muscle, skin, and fat were investigated. The transmitted and reflected spectra are shown in Fig. 8 for sheep and cow samples.

### IMN holes laser indicator in animal tests

Animal experiments were performed on 10 samples in two group, including 5 sheep legs and 5 cow legs. For each sample, intramedullary nailing performed inside the tibia bone, and distal locking was done three times using laser intramedullary nail holes detector. The output parameters were procedure time and drilling quality shows in Tables 1 and 2.

**Table 1:** The procedure times for each interlocking of IMN hole in sheep and cow samples.

Sample number	Procedure time (s)						P-value (α = 0.05)
	Hole.1	Hole.2	Hole.3	Mean	SD	Overall procedure time	
**Sheep**							
1	129	98	108	99.866	14.532	99.866 ± 14.532	P < 0.05
2	96	121	102				
3	92	104	88				
4	108	98	93				
5	88	64	94				
**Cow**							
1	222	94	80	120.333	33.047	120.333 ± 33.047	
2	146	132	99				
3	105	103	152				
4	123	104	106				
5	119	99	121

**Table 2:** The drilling quality points for each interlocking of IMN hole in sheep and cow samples.

Sample number	Drilling quality (points)						P-Value (α = 0.05)
	Hole.1	Hole.2	Hole.3	Mean	SD	Overall drilling quality	
**Sheep**							
1	3	3	2	2.733	0.457	2.733 ± 0.457	P > 0.05
2	3	2	3				
3	3	3	3				
4	2	3	3				
5	3	2	3				
**Cow**							
1	2	3	2	2.666	0.487	2.666 ± 0.487	
2	3	2	3				
3	3	3	3				
4	2	3	3				
5	3	3	2				

### IMN holes laser indicator in a human test

Intramedullary nailing was performed for the tibia bone on a middle-aged man. The IMN holes laser indicator used in the dark conditions of the surgery room to interlocking IMN. First, one hole was interlocked in the distal area, and then two holes were interlocked in the proximal area. The interlocking process was successful and there were no errors. The output parameters, the procedure time and the drilling quality are shown in Tables 3 and 4.

**Table 3:** The procedure time for each interlocking of IMN hole in the human test using the IMN holes laser indicator, Targeting-Arm Device (TAD), and Free-Hand (FH) techniques.

	Procedure time (s)			Mean	SD	Overall procedure time	P-value (α = 0.05)	
	Hole.1	Hole.2	Hole.3				IMN holes laser indicator and TAD	IMN holes laser indicator and FH
IMN holes laser indicator	128	110	108	115.333	8.993	115.333 ± 8.993	P < 0.05	P < 0.05
TAD	423	300	257	326.666	70.343	326.666 ± 70.343		
FH	932	897	921	916.666	14.613	916.666 ± 14.613

**Table 4:** The drilling quality points for each interlocking of IMN hole in the human test using the IMN holes laser indicator, Targeting-Arm Device (TAD), and Free-Hand (FH) techniques.

	Drilling quality (points)			Mean	SD	Overall drilling quality	P-value (α = 0.05)	
	Hole.1	Hole.2	Hole.3				IMN holes laser indicator and TAD	IMN holes laser indicator and FH
IMN Holes Laser Indicator	3	3	2	2.666	0.471	2.666 ± 0.471	P < 0.05	P = 0.05
TAD	1	1	3	1.666	0.942	1.666 ± 0.942		
FH	2	3	3	2.666	0.471	2.666 ± 0.471		

### TAD in a human test

Four fixation screws were inserted in the IMN using the TAD. The distal interlocking process encountered errors while the proximal interlocking performed successfully. For the first and second holes at the distal area of the nail, the drilling position was changed 4 and 3 times, respectively. While for two holes in the proximal area of the nail, the drilling process was performed while the drill did not hit the nail. The output parameters are shown in Tables 3 and 4.

### FH technique in a human test

Interlocking of the IMN was done by four fixation screws using a c-arm device. The movement direction of the drill was controlled under X-ray emission. So, no severe interference occurred between the drill and IMN. But the procedure time was increased comparing using the IMN holes laser indicator device and TAD. Although the surgery team was used X-ray protective lead clothes with a thyroid shield collar, the X-ray emission isn`t ineffective on their health especially the patient. The output parameters, the procedure time and the drilling quality are shown in Tables 3 and 4, respectively.

## Discussion

According to the results of spectroscopy of biological tissues, for higher wavelength, higher transmittance is obtained. Due to the white colour of bone, a uniform reflectance is observed on the visible spectral range. Regards to the denser bone of cow, it illustrates lower transmittance. Two dips in wavelengths of 540 nm and 576 nm with a distance of 36 nm in the reflectance spectrum of sheep tendon and two dips in wavelengths of 490 nm and 536 nm, with a distance of 46 nm in the reflectance spectrum of cow samples are observed. These dips can be related to the effect of blood absorption on the endogenous fluorescence signal intensity of biological tissues^41,43^. Changes amount of blood in the tendon vessels can affect the fluorescence spectra^44,45^. It may result from α and β attenuation signals due to the absorption capacity of the hemoglobin concentration^46,47^. Moreover, different amounts of fluorescent coenzyme concentration in cow and sheep samples have a great effect on the transmitted intensity. The muscle transmitted and reflected spectra are clearly related to its dark red color. The sample of sheep`s skin has a lighter brown color than cows according to its reflectance diagrams. Fat also illustrates an almost flat reflectance, like the bone spectrum regards to its white color.

Therefore, the optimized wavelength of 680 nm is selected for the IMN holes laser indicator. In addition to low transmittance in lower wavelengths and thus a mitigated performance of the device, the highest absorbance makes these wavelengths unsafe for tissues and organs.

There is a significant difference in the overall procedure time (sheep legs = 99.866 ± 14.532 s vs. cow legs = 120.333 ± 33.047 s, P < 0.05) between the two groups. But there isn`t a significant difference in drilling quality (sheep legs = 2.733 ± 0.457 points vs. cow legs = 2.666 ± 0.487 points, P > 0.05) between the two groups. The overall procedure time obtained for sheep samples compared to the cow samples is less. It`s due to the larger dimensions of the cow's leg than the sheep's. Cow's leg has larger dimensions and thicker, denser tissues than sheep's leg. So cutting tissues and drilling the bone procedures takes more time. If we compare the sheep's leg to an immature human's leg being, and the cow's leg to an adult human's leg being. It can be said that the nailing procedure for an adult compared to an immature human takes more time.

Weidert et al., during an intramedullary nailing study performed on cow legs, reported 378.76 ± 101.10 s for conventional fluoroscopy (free hand technique), and 380.38 ± 165.11 s for video-augmented fluoroscopy^41^. The overall procedure time on cow samples obtained 120.333 ± 33.047 s using the IMN holes laser indicator. The time obtained is about 260.047 s less than the video-augmented fluoroscopy, and about 258.427 s less than the conventional fluoroscopy. Using the IMN holes laser indicator, the procedure time can be reduced compared to fluoroscopy techniques. It`s an X-ray-free technique. While in free hand technique, the patient and the surgical team are exposed to X-ray radiation. However, the fluoroscopy technique can evaluate the reliability and validation of the fracture healing methods^48–50^.

There aren`t any significant differences in drilling quality between sheep and cow samples (p > 0.05). For sheep samples, 11 holes interlocked successfully without drill impact with the nail, and 4 holes interlocked successfully with a slight collision with the nail. Moreover, in cow samples, 10 holes interlocked successfully without drill impact with the nail, and 5 holes interlocked successfully with a slight collision with the nail. This small difference may be due to human error due to experimenter fatigue of multiple tests. Slight collision with the nail is normal and does not interfere with the process.

By comparing the overall procedure times of the IMN holes laser indicator, and the TAD techniques in the human tests, it is clear the IMN holes laser indicator significantly takes less time to interlock the IMN (IMN holes laser indicator technique = 115.333 ± 8.993 s vs. TAD technique = 326.666 ± 70.343 s, P < 0.05). Also, there is a significant difference between the IMN holes laser indicator, and the FH techniques (IMN holes laser indicator technique vs. FH technique = 916.666 ± 14.613 s, P < 0.05). There is significant difference in drilling quality between the IMN holes laser indicator, and the TAD techniques (the IMN holes laser indicator technique = 2.666 ± 0.471 points vs. the TAD technique = 1.666 ± 0.942 points, P < 0.05). Moreover, the drilling quality between the IMN holes laser indicator, and FH techniques is equal (the IMN holes laser indicator technique vs. the FH technique = 2.666 ± 0.471 points, P = 0.05).

As shown in Table 5, there are different reports of the IMN interlocking in human tests which use the TAD, and the FH techniques. Among these, Ramireddy et al. have reported less procedure time for the IMN interlocking by the TAD technique (180–300 s)^62^. Also, Windolf et al., have reported less procedure time for the FH technique (246 ± 126)^39^. Comparing the reported times with the obtained time for the IMN holes laser indicator, it is obvious that the IMN holes laser indicator technique takes less procedure time (115.333 ± 8.993).

**Table 5:** Different reported procedure times for the free hand technique (FH) and the targeting-arm devices (TAD).

Author	Journal	Fracture type	Procedure time (s)		Reference number
			FH	TAD	
Krettek, C. et al	JOT 1997	Tibia	–	930	^51^
Krettek, C. et al	JOT 1998	Tibia	1314 ± 630	1002 ± 516	^52^
Pardiwala, D. et al	Injury 2001	Femoral	2148 ± 1116	1158 ± 588	^53^
Gugala, Z. et al	Injury 2001	Tibia	1144.8	1023.6	^54^
Suhm, N. et al	Injury 2004	Femoral and Tibial	822 ± 282	–	^22^
Anastopoulos, G. et al	CORR 2008	Tibia	–	390 ± 126	^10^
Anastopoulos, G. et al	Injury 2008	Femoral	–	396 ± 156	^55^
Arlettaz, Y. et al	Injury 2008	Tibia	–	1440	^56^
		Femoral	–	1860	
Rohilla, R. et al	Intl Orthop 2009	Femoral	–	1460.4 ± 362.4	^57^
Windolf, M. et al	BMC Musculoskelet Disord 2012	Tibia	246 ± 126	–	^39^
Maqungo, S. et al	J Orthop Trauma 2014	Femoral	600	–	^58^
Han, B. et al	Medicine (Baltimore) 2017	Femoral	1170 ± 360	–	^59^
Wang, Y. et al	Medicine 2018	Tibia	735.6 ± 264	–	^36^
Seyhan, M. et al	Cerrahpaşa Med J 2020	Tibia	1290 ± 348	–	^60^
Ramireddy, M. et al	Int J Orthop Sci 2020	Tibia	408	180–300	^61^
Gao, H. et al	Int J Gen Med 2021	Femoral	212 ± 105	–	^62^

**Figure 9:**
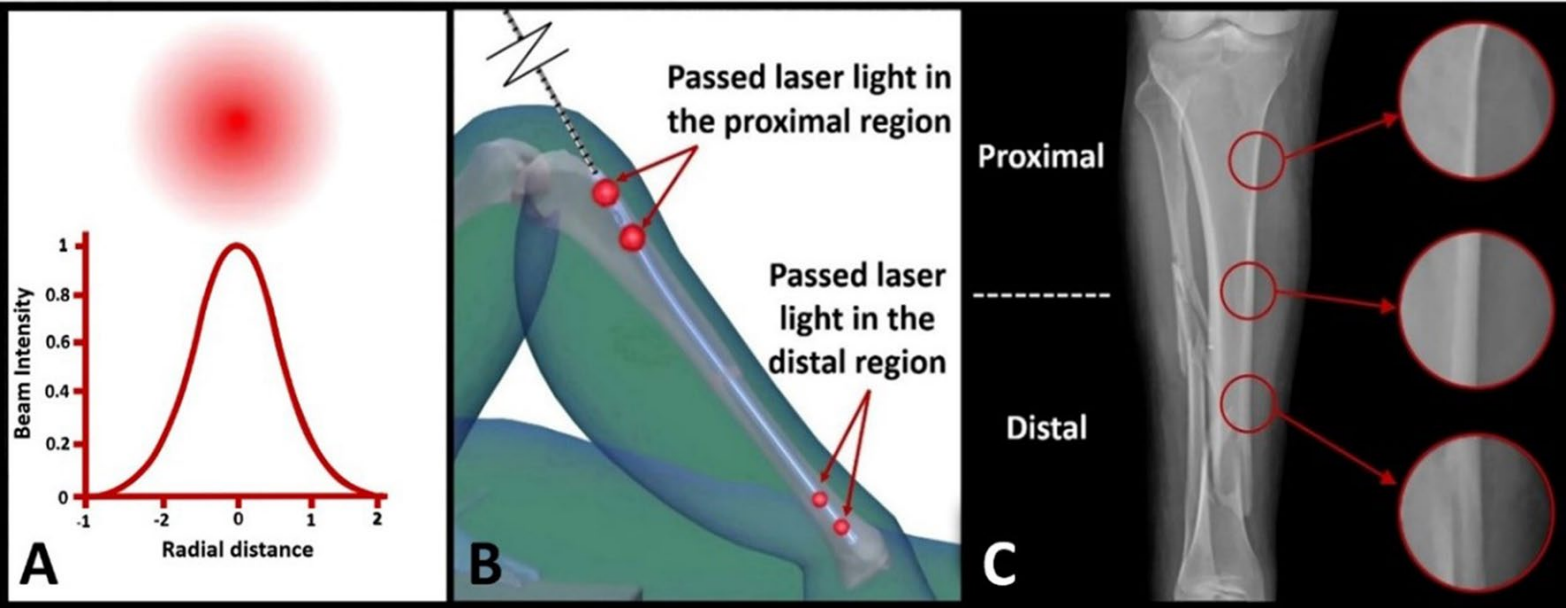
(**A**) The Gaussian intensity profile of the laser beam, (**B**) the schematic image of the light passing through the nail holes in proximal and distal regions and (**C**) the cortex of the patient tibia bone in three regions before the surgery.

Transmitted light intensity through soft tissues such as fat is more than hard tissues like bone (as shown in Fig. 8). This reduces the intensity of the transmitted light through the patient's foot in areas with thicker soft tissue. Regarding the Gaussian intensity profile of the laser beam (Fig. 9A), in a plane perpendicular to the beam axis, the emitted beam from the laser is more intense in the center and decreases in a Gaussian shape^63^. Therefore, the transmitted beam through the patient's foot in the center is more intense, which makes it easier to detect the center of the circle created by the laser beam on the patient's foot. This prevents errors in determining the center position of the IMN hole placed in the patient's foot. Also, the shape of the soft tissue layers on the bone is almost identical, which has no effect on the accuracy of the system.

During evaluating the IMN holes laser indicator in a human test, by inserting the probe inside the IMN, the light passing through the nail holes in the proximal region was more intense than in the distal region as shown in schematic image Fig. 9B. Moreover, the diameter of the circle created on the patient's foot due to the passing of light in the distal was smaller than the proximal. However, the diameter of the foot is larger in the proximal, and we should have seen less light passing through this region. As shown in Fig. 9C, the cortex in the proximal of the tibia bone has a small thickness and a large diameter. Approaching the distal, the thickness of the cortex increases, and the diameter of the tibia bone decreases. Therefore, the tibia bone is strong enough in a small diameter area. The cortex is denser than other parts of the bone. The passage of the beam through the dense area is more difficult than the less dense area. As a result, as approaching the distal of the tibia, the passage of the beam decreases so is observed a circle with a smaller diameter in the distal. By reducing the diameter of the circle, the position of the nail hole can be determined more accurately.

## Conclusion

IMN holes laser indicator is a non-invasive accurate method that reduced surgical time and simplifies the process. This is an X-ray-free technique and prevents patient and surgical team from being exposed to X-ray. This new technology makes it easier to determine the drilling position and interlocking IMN especially distal interlocking which is a big problem for orthopedic surgeons. This new technology uses 680 nm visible light which has optimal transmission, reflectance, and absorption properties that can be seen with the naked eye. This instrument paves the way for development of effective and safe intramedullary nailing in clinical applications.

## References

[1] Foote CJ (2015). Which surgical treatment for open tibial shaft fractures results in the fewest reoperations? A network meta-analysis. Clin. Orthop. Relat. Res..

[2] Ricci WM, Gallagher B, Haidukewych GJ (2009). Intramedullary nailing of femoral shaft fractures: Current concepts. JAAOS..

[3] Lanzetti RM (2018). Comparison between locked and unlocked intramedullary nails in intertrochanteric fractures. Eur. J. Orthop. Surg. Traumatol..

[4] Bisaccia, M. et al. Feasibility and value of non-locking retrograde nail vs. locking retrograde nail in fixation of distal third femoral shaft fractures: Radiographic, bone densitometry and clinical outcome assessments. *Medicinski Glasnik*. **17**, 163–169 (2020). 10.17392/1097-20 31994857

[5] Basal O, Kırdemir V, Baykal B (2018). Accuracy of distal long femur nail locking with different techniques. Biomed. J. Sci. Tech. Res..

[6] Ma JX (2017). Comparison of clinical outcomes with InterTan vs Gamma nail or PFNA in the treatment of intertrochanteric fractures: A meta-analysis. Sci. Rep..

[7] White, N. J., Sorkin, A. T., Konopka, G. K. & McKinley, T. O. Surgical technique static intramedullary nailing of the femur and tibia without intraoperative fluoroscopy*. Clin. Orthop. Relat. Res*. **469**, 3469–3476 (2011). 10.1007/s11999-011-1829-7 PMC3210293 21369767

[8] Hussain N (2016). Intramedullary nailing versus plate fixation for the treatment displaced midshaft clavicular fractures: A systematic review and meta-analysis. Sci. Rep..

[9] Krettek C (1998). Deformation of femoral nails with intramedullary insertion. J. Orthop. Res..

[10] Anastopoulos G, Ntagiopoulos PG, Chissas D, Papaeliou A, Asimakopoulos A (2008). Distal locking of tibial nails a new device to reduce radiation exposure. Clin. Orthop. Relat. Res..

[11] Diotte B (2012). Radiation-free drill guidance in interlocking of intramedullary nails. MICCAI..

[12] Fernandez, A. A. Coplanar X-ray guided aiming arm for locking of intramedullary nails. *United States Patent*. Patent No: US 7481,815 B2 (2009).

[13] Lerner, A., Nassonov, A. & Diamant, L. System and method for locating of distal holes of an intramedullary nail. *United States Patent*. Patent No: US 8,231,629 B2 (2012).

[14] Kienzle, T. C. Computer assisted intramedullary rod surgery system with enhanced features. *United States Patent.* Patent No: US 2005/0251113 A1 (2005).

[15] Zheng, G. & Zhang, X. Method and device for computer assisted distal locking of intramedullary nails. *United States Patent*. Patent No: US 8.444,645 B2 (2013).

[16] Koutenaei BA (2019). Radiation-free methods for navigated screw placement in slipped capital femoral epiphysis surgery. IJCARS..

[17] Yoo JI (2019). Comparison of intraoperative radiation exposure with and without use of distal targeting device: A randomized control study. Arch. Orthop. Trauma Surg..

[18] Yiannakopoulos CK, Kanellopoulos AD, Apostolou C, Antonogiannakis E, Korres DS (2005). Distal intramedullary nail interlocking the flag and grid technique. J. Orthop. Trauma.

[19] Suhm N, Jacob AL, Nolte LP, Regazzoni P, Messmer P (2000). Surgical navigation based on fluoroscopy-clinical application for computer-assisted distal locking of intramedullary implants. Comput. Aided Surg..

[20] Leloup T, Kazzi WE, Schuind F, Warzee N (2008). A novel technique for distal locking of intramedullary nail based on two non-constrained fluoroscopic images and navigation. IEEE Trans. Med. Imaging.

[21] Zheng G (2008). A robust and accurate two-stage approach for automatic recovery of distal locking holes in computer-assisted intramedullary nailing of femoral shaft fractures. IEEE Trans. Med. Imaging.

[22] Suhm N, Messmer P, Zuna I, Jacob LA, Regazzoni P (2004). Fluoroscopic guidance versus surgical navigation for distal locking of intramedullary implants. A prospective, controlled clinical study. Injury.

[23] Endo, M., Nakajima, H., Arao, M. & Hata, Y. Eddy current system for finding distal transverse screw holes of an intramedullary nail. *IEEE 2006 World Automation Congress,* Budapest, Hungary (2006).

[24] Lei H, Sheng L, Manyi W, Junqiang W, Wenyong L (2010). A biplanar robot navigation system for the distal locking of intramedullary nails. Int. J. Med. Robot. Comput. Assist. Surg..

[25] Junejo F, Marouf KB, Kerr D, Taylor AJ, Taylor GJS (2007). X-ray-based machine vision system for distal locking of intramedullary nails. Proc. Inst. Mech. Eng..

[26] Liao H (2004). Surgical navigation by autostereoscopic image overlay of integral videography. IEEE Trans. Inf Technol. Biomed..

[27] Liao H, Inomata T, Sakuma I, Dohi T (2010). 3-D augmented reality for MRI-guided surgery using integral videography autostereoscopic image overlay. IEEE Trans. Biomed. Eng..

[28] Wang J (2014). Augmented reality navigation with automatic marker-free image registration using 3-D image overlay for dental surgery. IEEE Trans. Biomed. Eng..

[29] Zhang X, Chen G, Liao H (2017). High quality see-through surgical guidance system using enhanced 3D autostereoscopic augmented reality. IEEE Trans. Biomed. Eng..

[30] Feuerstein M, Reichl T, Vogel J, Traub J, Navab N (2009). Magneto-optical tracking of flexible laparoscopic ultrasound: Model-based online detection and correction of magnetic tracking errors. IEEE Trans. Biomed. Eng..

[31] Qi, Y., Sadjadi, H., Yeo, C. T., Zaad, K. H. & Fichtinger, G. Electromagnetic tracking performance analysis and optimization. *36th Annual International Conference of the IEEE Engineering in Medicine and Biology Society*, Chicago, IL, 6534–6538 (2014). 10.1109/EMBC.2014.6945125 25571493

[32] Hoffmann M (2012). Next generation distal locking for intramedullary nails using an electromagnetic X-ray-radiation-free real-time navigation system. J. Trauma Acute Care Surg..

[33] Choi J (2017). A novel smart navigation system for intramedullary nailing in orthopedic surgery. PLoS ONE.

[34] Wong TH (2017). Novel passive two-stage magnetic targeting devices for distal locking of interlocking nails. J. Healthc. Eng..

[35] Ma, L. *et al*. Three‐dimensional augmented reality surgical navigation with hybrid optical and electromagnetic tracking for distal intramedullary nail interlocking. *Int. J. Med. Robot. Comput. Assist. Surg*. 10.1002/rcs.1909 (2018). 10.1002/rcs.1909 29575601

[36] Wang, Y. *et al*. Comparison of free-hand fluoroscopic guidance and electromagnetic navigation in distal locking of tibia intramedullary nails. *Medicine*. 10.1097/MD.0000000000007450 (2017). 10.1097/MD.0000000000011305 PMC6076088 29979399

[37] Thomas, J. R. Apparatus and method for implanting an intramedullary rod. *United States Patent.* Patent No: 5,127,913 (1992).

[38] Trecha, R. R. Coaxial laser targeting device for use with x-ray equipment and surgical drill equipment during surgical procedures. *United States Patent*. Patent No: 5,031,203 (1991).

[39] Windolf, M. *et al*. Reinforcing the role of the conventional C-arm: A novel method for simplified distal interlocking. *BMC Musculoskelet. Disord*. (2012). 10.1186/1471-2474-13-8. 10.1186/1471-2474-13-8 PMC3305668 22276698

[40] Navab N, Heining SM, Traub J (2010). Camera augmented mobile C-Arm (CAMC): Calibration, accuracy study, and clinical applications. IEEE Trans. Med. Imaging.

[41] Weidert, S. *et al*. Video‐augmented fluoroscopy for distal interlocking of intramedullary nails decreased radiation exposure and surgical time in a bovine cadaveric setting. *Int. J. Med. Robot. Comput. Assist. Surg*. 10.1002/rcs.1995 (2019). 10.1002/rcs.1995 30861265

[42] Diotte B (2015). Multi-modal intra-operative navigation during distal locking of intramedullary nails. IEEE Trans. Med. Imaging.

[43] Dremin, V. V. *et al*. The development of attenuation compensation models of fluorescence spectroscopy signals. *SPIE Third International Symposium on Optics and Biophotonics and Seventh Finnish-Russian Photonics and Laser Symposium,* Saratov, Russia (2016).

[44] Zonios, G., Bykowski, J. & Kollias, N. Skin, Melanin. Hemoglobin, and light scattering properties can be quantitatively assessed in vivo using diffuse reflectance spectroscopy. *J. Invest. Dermatol*. **117**, 1452–1457 (2001). 10.1046/j.0022-202x.2001.01577.x 11886508

[45] Fenwick SA, Hazleman BL, Riley GP (2002). The vasculature and its role in the damaged and healing tendon. Arthritis Res..

[46] Edwards P (2017). Smartphone based optical spectrometer for diffusive reflectance spectroscopic measurement of hemoglobin. Sci. Rep..

[47] Zonios G (1999). Diffuse reflectance spectroscopy of human adenomatous colon polyps in vivo. Appl. Opt..

[48] Maiettini D (2016). Feasibility and value of radiographic union score hip fracture after treatment with intramedullary nail of stable hip fractures. Acta Inform. Med..

[49] Rollo G (2019). Radiographic, bone densitometry and clinic outcomes assessments in femoral shaft fractures fixed by plating or locking retrograde nail. Med Arch..

[50] Meccariello, L. *et al*. Locking retrograde nail, non‑locking retrograde nail and plate fixation in the treatment of distal third femoral shaft fractures: Radiographic, bone densitometry and clinical outcomes. *J. Orthop. Traumatol*. **22**, (2021). 10.1186/s10195-021-00593-9 PMC8339178 34350532

[51] Krettek C (1997). A new technique for the distal locking of solid AO unreamed tibial nails. JOT..

[52] Krettek C (1998). Experimental study of distal interlocking of a solid tibial nail: Radiation-independent distal aiming device (DAD) versus freehand technique (FHT). JOT..

[53] Pardiwala D (2001). The AO distal locking aiming device: An evaluation of efficacy and learning curve. Injury.

[54] Gugala Z (2001). Tibial intramedullary nail distal interlocking screw placement: Comparison of the free-hand versus distally-based targeting device technique. Injury.

[55] Anastopoulos G (2008). Distal locking of tibial nails a new device to reduce radiation exposure. Clin. Orthop. Relat. Res..

[56] Arlettaz Y (2008). Targeting device for intramedullary nails: A new high-stable mechanical guide. Injury.

[57] Rohilla R (2009). Nail over technique for distal locking femoral intramedullary nails. Int. Orthop..

[58] Maqungo S (2014). Distal interlocking screw placement in the femur: Free-hand versus electromagnetic assisted technique (sureshot). J. Orthop. Trauma.

[59] Han, B. *et al*. Comparison of free-hand fluoroscopic guidance and electromagnetic navigation in distal locking of femoral intramedullary nails. *Medicine (Baltimore)*. **96**, e7450 (2017). 10.1097/MD.0000000000007450 PMC5521895 28723755

[60] Seyhan M (2020). A new distal locking technique in intramedullary nailing. Cerrahpaşa Med. J..

[61] Ramireddy M (2020). A study on application of newly designed device for targeting second interlocking hole in tibial fractures. Int. J. Orthop. Sci..

[62] Gao H (2021). A new accurate, simple and less radiation exposure device for distal locking of femoral intramedullary nails. Int. J. Gen. Med..

[63] Steen, W. M. & Mazumder, J. *Laser Material Processing.* 4th edition. Springer (2010).

